# Systematic Neighborhood Observations at High Spatial Resolution: Methodology and Assessment of Potential Benefits

**DOI:** 10.1371/journal.pone.0020225

**Published:** 2011-06-03

**Authors:** Tammy C. M. Leonard, Margaret O'Brien Caughy, Judith K. Mays, James C. Murdoch

**Affiliations:** 1 School of Economic, Political and Policy Sciences, University of Texas at Dallas, Richardson, Texas, United States of America; 2 University of Texas School of Public Health, Dallas, Texas, United States of America; Yale University School of Medicine, United States of America

## Abstract

There is a growing body of public health research documenting how characteristics of neighborhoods are associated with differences in the health status of residents. However, little is known about how the spatial resolution of neighborhood observational data or community audits affects the identification of neighborhood differences in health. We developed a systematic neighborhood observation instrument for collecting data at very high spatial resolution (we observe each parcel independently) and used it to collect data in a low-income minority neighborhood in Dallas, TX. In addition, we collected data on the health status of individuals residing in this neighborhood. We then assessed the inter-rater reliability of the instrument and compared the costs and benefits of using data at this high spatial resolution. Our instrument provides a reliable and cost-effect method for collecting neighborhood observational data at high spatial resolution, which then allows researchers to explore the impact of varying geographic aggregations. Furthermore, these data facilitate a demonstration of the predictive accuracy of self-reported health status. We find that ordered logit models of health status using observational data at different spatial resolution produce different results. This implies a need to analyze the variation in correlative relationships at different geographic resolutions when there is no solid theoretical rational for choosing a particular resolution. We argue that neighborhood data at high spatial resolution greatly facilitates the evaluation of alternative geographic specifications in studies of neighborhood and health.

## Introduction

In the last 15 years, public health researchers have documented disparities in health status associated with the structural and social characteristics of neighborhoods that cannot be explained by individual differences in risk profiles. A broad range of health outcomes has been considered in neighborhood research including indices of adult physical health [1], [2], [3], [4], [5], [6], [7], [8], [9], adult mental health [10], [11], [12], [13], [14], [15], [16], and child health [6], [7], [17], [18], [19], [20], [21], [22], [23], [24], [25], [26], [27], [28].

Health outcome data at smaller geographic resolution (for example spatially referenced individual level data) are becoming increasingly available, furthering the study of neighborhood effects on health. Unfortunately, secondary data sources, such as the census, may be inadequate for these studies because aggregating over census geographies (e.g. blockgroups, tracts, etc) loses much of the variation that is valuable for analysis of individual level data. To address the limitations of secondary data sources, a number of public health researchers have employed community audits to provide not only current data on neighborhoods but also direct observation of neighborhood conditions.

Two recent reviews of the use of neighborhood observation methods in public health research [29], [30] document the wide range of approaches used in the implementation of community audit methods, and the limitations of the extant literature on these methods. Spatial resolution is one of the key factors along which built environment data differ. There are many reasons for this. First, if secondary data is used, the researcher must adjust and use the best spatial resolution available. In the absence of other available data, some information about the built environment is certainly better than none and some policy questions can be broadly assessed without high-resolution data. Second, data at higher spatial resolution is more expensive to obtain because a larger number of observations per area are needed. Costs and benefits of collecting and analyzing high-resolution vs lower resolution observational data should be considered. Additionally, the scale of the research question sometimes determines the resolution of data used. It is impractical for researchers who are studying the impact of a nation or state wide program to attempt to use the method presented here to collect high-resolution observational data. However, research studies focusing on smaller geographies (within a neighborhood or a city –sector) might find high-resolution data more beneficial. In short, the research question should determine the spatial resolution needed.

With this in mind, there is a need to examine the reliability and predictive utility of observational data collected at varying levels of aggregation. Examining the robustness of the relationship between individual outcome measures and observational data at different geographic aggregation levels is essential for investigating the presence of potential omitted variable biases that result when geographic boundaries are correlated with unobserved individual characteristics. Additionally, policy initiatives that operate at varying geographic scope would be better informed if the robustness of results to lower (and higher) level aggregation schemes were evaluated. However, due to the level of spatial aggregation at which the observational data are often collected, most of the existent data sets are not well suited for a thorough investigation of this robustness. In this paper, we present a new interdisciplinary community audit methodology for collecting data on neighborhood factors at the smallest geographic unit possible and assess the advantages of using data at such a high spatial resolution for micro-level studies in neighborhood-related health outcomes.

Previous work in this arena, such as the extensive data collected in conjunction with the Project on Human Development in Chicago Neighborhoods (PHDCN) and many others, has focused on the face block—or the parcels facing the same street segment or “block”– as the smallest geographic level for collecting observational data, but our methodology increases the spatial resolution by analyzing the parcel. In residential neighborhoods, a parcel consists of the house and surrounding yard or all of the property that a homeowner owns and is assessed for tax purposes. Most neighborhood audit tools measure some elements of both the physical and social conditions in the neighborhood. The parcel observation methodology we present focuses exclusively on the physical dimension. Raudenbush and Sampson [31] analyzed PHDCN data and find that measures of physical disorder are relevant gauges of neighborhood condition at higher spatial resolution than measures of social disorder. Further, they note that reliability among face block measures of physical disorder may be improved by increasing the number of items observed for each face block. Collecting parcel observations achieves this goal. Beyond the ecometrics-based rational put forth by Raudenbush and Sampson [31], there are also numerous practical reasons for expanding data collection to include parcel observations: (1) it allows for maximum flexibility with regards to spatial aggregation; (2) it allows the researcher to distinguish between observations which the household has direct control (i.e. the upkeep of their yard/property) and observations which are impacted by others in the community (i.e. the upkeep of common areas such as parks or the upkeep of other properties in the neighborhood); and (3) it allows for the data and research outcomes to be related to property values which have direct policy impact through the tax base. However, while the advantages appear strong, no systematic work has been done to determine whether the advantages outweigh the costs of collecting data at this micro level.

An issue related to geographic aggregation is the problem of how to operationally define neighborhood boundaries—or put more generally, the modifiable areal unit problem (MAUP) [32]. When addressing the question about how to measure neighborhood characteristics, Guo and Bhat [33] state that “we should measure what matters to people over the area that really matters to people” (p. 31). This suggests neighborhood boundaries should be selected thoughtfully and may vary depending upon the research question at hand. To date, a few investigators studying the effects of neighborhood context on health have utilized sophisticated spatial definitions of neighborhoods [34], [35], [36], [37], [38], but no studies have been able to comprehensively compare the utility of community audit data collected at varying levels of aggregation. This is a significant gap in the extant literature. Boone et al [39] find that associations between physical activity and street connectivity vary by setting and geographic scale. The same is likely true of associations between health outcomes and neighborhood observational data. Most often, however, public health researchers rely upon administrative boundaries of neighborhoods such as census tracts or census block groups, and only a single geographic scale is analyzed.

The observational instrument developed is intended to be useful for analyzing the relationship between place and individual health and well-being while avoiding biased created by the MAUP. Can data at a high spatial resolution improve studies and, in particular, public health policy implications regarding the relationship between neighborhood conditions and health? While we acknowledge that data at lower spatial resolution is helpful and sufficient in some cases, we believe that higher resolution does present advantages that can improve and fine-tune policy implications. First, data at high spatial resolution allow the researcher nearly complete flexibility in specifying neighborhood boundaries and hence a thorough investigation of MAUP-bias is possible (though we note that a thorough analysis of this issue is outside the scope of this paper). For example, studies may find a statistically significant correlation between average census tract condition and obesity. However, unless one investigates this relationship further at varying levels of geographic resolution, it is unknown if the results are biased by other omitted variables correlated with the geographic census tract definition. Second, data at high spatial resolution allow public health policy makers to identify the most appropriate geographic level for public health interventions. Continuing with the previous example, a relationship between census tract condition and health is an important observation, but policy relevance may be greatly improved if additional insight was available on the geographic scale that scarce public resources should be deployed to improve public health. Policy makers need to know the comparative implications for enacting, for example, broad-based local neighborhood clean-up initiatives throughout the census tract, versus concentrated initiatives to improve only the most blighted areas within the census tract.. Neighborhood observational data will allow the research to specify exactly which geographic definitions matter most for a particular policy implication.

The purpose of this report is three-fold. First, we describe the methodology and how the method was implemented to ensure the collection of high quality observational data. Next, we analyze the reliability of the data and the relationships found between the variables observed. Finally, we examine the utility of these data to examine neighborhood conditions at different levels of aggregation and how such data might be used in studies of neighborhoods and health.

## Methods

### Ethics Statement

All research involving human subjects has been approved by the Institutional Review Board of the University of Texas at Dallas (IRB Approval Number 08-33). Informed written consent for study participation was obtained from each human subject. The consent forms used were approved by the IRB committee.

### Study design

The new observational method was used as part of a large cross-sectional-longitudinal research project aimed at studying the effects of publicly driven investment in a low-income, minority neighborhood. The neighborhood examined, commonly known as Fair Park, is an area of over 2000 acres with approximately 20,000 residents located less than two miles southeast of downtown Dallas, Texas. The primary reason for research interest in Fair Park is that this community received a large injection of publicly-directed investment beginning in 2009. In particular, the Dallas Area Rapid Transit (DART) authority has built a new light rail line into Fair Park and approximately $80M has been allocated for housing and infrastructure development in the neighborhood.

The neighborhood consists of thirty-two block groups falling into seven census tracts. The area is poor (median household income is $19,939) and primarily African American (70%), although the percentage of the population that is Hispanic (26%) has been growing (estimates based on US Census, 2005–2009 American Community Survey 5 Year Estimates). The study area contains primarily single-family residential housing of which approximately 52% are owner occupied.

In addition to the neighborhood observational data, a short door-to-door survey was conducted to collect basic demographic data from neighborhood residents. The 1210 households included in this survey were sampled according to a geographically weighted sampling scheme with oversampling of residents residing near the new neighborhood light rail stations. The sampling scheme was designed to facilitate a longitudinal study analyzing the impact of light rail investment in the neighborhood. Included in the survey was self-reported health status in which residents were asked to rate their physical health status as excellent, very good, good, fair or poor. Self-reported health status will be used to provide some indication of the association between neighborhood observational measures and health.

### Observational instrument

The instrument used for the systematic neighborhood observations was based on the integration of the Neighborhood Observation Checklist (NOC) [40] and existing coding methodologies used by the City of Dallas which have their history in the urban planning literature. The resulting methodology allows for observations at the parcel level and provides data relevant to the study of public health, economics, urban planning and other social science disciplines. As stated in the introduction, one of the particular ways in which parcel level observations differ from lower spatial resolution neighborhood data (e.g. faceblock, census blockgroup, cenus tract) is that individuals have great control over their own parcel condition while control over other neighborhood attributes is limited. Thus, the parcel observation has two roles: it is the building block from which lower spatial resolution observations are built and it provides individual data for the parcel's occupant. Items for the observation are described in Table 1. Some items were characterized at the parcel level, and some were characterized at the face block level. As previously noted, the primary innovation of the method is the data collected at the parcel level, which includes only measures of physical condition (as opposed to social conditions). Additionally, parcel usage data was collected for all parcels in the neighborhood while the other parcel condition codes were observed only for single-family residence parcels.

**Table 1:** Systematic social observational items at the parcel and face block level.

*Items observed at the parcel level*	
Item	Description
Parcel usage	See Appendix for complete list of parcel use codes
Incompatibility of land use	Yes if incompatible (e.g., residential next to junk yard or a vacant lot)
Area	Square feet
Condition of house	Good, cosmetic repairs, structural repairs, tear down condition
Peeling paint	Yes if present
Broken windows	Yes if present
Boarded windows	Yes if present
Barred windows	Yes if present
Barred doors	Yes if present
Uncovered crawl space	Yes if present
Condition of lawn	Well-kept or unkempt
Condition of fence	Well-kept or in poor shape
Trash on curb	Yes if bulk trash along the curb
Trash in yard	Yes if bulk trash or junk in the yard
Cars in yard/drive	Yes if vehicles in need of repair in the yard/drive
City citation (yard)	Yes if city code enforcement sign in the yard
City citation (house)	Yes if city code enforcement sign in window

*Items observed at face block level*	
Item	Description
Street width	Number of lanes
Divided street	Coded yes if divided by median
Street condition	Rough, average, excellent
Sidewalk	Both sides, one side, none
Sidewalk condition	Rough, average, excellent
Curbs	Both sides, one side, none
Street lamps	None, one, two or more
Alley	Both sides, one side, none
Trees	Yes if a tree-lined street
Congested	Yes if insufficient room for two cars to pass
Children	Yes if 2+ children observed playing
Adults	Yes if 2+ adults gathered outside
Dogs	Yes if dogs not on leashes or secured in fences
Overall impression	Desirable or undesirable

Basemaps and geodatabases were built using spatial data downloaded from the North Central Texas Council of Governments (NCTCOG, http://clearinghouse.dfwmaps.com/) to which parcel data from the Dallas Central Appraisal District (DCAD) were added. Custom data input forms were created so that data could be entered directly into laptop and tablet computers running *ArcPad* software (ESRI, www.esri.com). Data collection staff could select a parcel or a block by clicking on the map and the appropriate data entry form would open for coding observations.

### Training of data collection staff

Data collection staff was recruited from the local community and trained in several classroom-based sessions. During these sessions, the staff was trained in the operation of the data collection software, the definitions of parcel and block attributes to be coded, and the procedures for conducting the observations. Supervisors and data collections conducted practice coding sessions in a comparable neighborhood. Data collectors were considered proficient when they had general agreement with themselves and the supervisors.

### Data collection methods

Pairs of trained data collectors collaborated to record observations of the attributes for each parcel and each block within the study boundary. One member of the pair drove the vehicle, while the other member operated the computer. On some residential streets, they were able to drive slowly while making the observations, while on busier commercial streets or when discussing their observations, they would park briefly. All codes were arrived at by consensus between the two observers. The observations were conducted during January 2009 and February 2009, and all observations were conducted on a weekday between the times of 9:00 a.m. and 4:30 p.m. Over a five week period, a total of 11,552 parcel and 1,778 face block observations were completed by varying pairs of 18 data collection staff.

### Quality assurance procedures of observational data

Several checks were instituted to assure data quality. First, the data collection supervisor periodically rode along with each data collection team and completed a checklist verifying the following: the observed parcel or block matched the ArcPad map, all attributes were noted, parcel use and face block condition were correctly coded, and no parcels or face blocks were missed. Second, the pairing of computer operator and driver was systematically varied to improve consistency of observations. Third, approximately 10% of each day's parcel and block observations were selected and coded a second time by a different pair of observers. This provides a set of independent observations from which inter-rater reliability can be calculated (see Results section). The two field visits for the 10% oversampling occurred at different times of day, but Raudenbush and Sampson [31] note that temporal variation presents little problem for observations of physical condition—the focus of the parcel data being analyzed. Finally, parcel use codes were checked against property tax codes obtained from the Dallas Central Appraisal District (DCAD) for potential discrepancies; all discrepancies were verified by a second field visit.

### Data processing of observational data

Parcel and block observations were retrieved daily from each field computer and merged into the appropriate geodatabase in ESRI's ArcGIS, also ensuring that each day's work would be saved. Maps were made showing the observed parcels and blocks. Using ArcMap tools, parcels were associated with block(s) that abutted the lot, and fields added to the parcel file specifying the adjacent block IDs. This process enabled the selection of parcels fronting both sides of a block, which, together with the street, made up the face block. Each parcel was also assigned to the proper census geographies (block, block group, and tract).

### Data collection costs

The primary cost of collecting observational data may be allocated to the following categories: (1) equipment, (2) GIS database creation and preparation, (3) data checking and supervision of field staff, (4) observers time in the field—including travel to and from locations and (5) the time required for data input after field observations have been made. Equipment used for this study includes ARC GIS software, 3 laptop or tablet PC's, and 1 supervisor PC. However, these are one time costs and often this equipment can be leveraged from other projects. The GIS database utilized for this work was adapted from the local tax appraiser's GIS database. While this information is not readily available in all communities, most urban areas do maintain GIS databases. GIS staff time required to assemble the GIS database and maps, prepare data entry forms, and load data and maps onto field computers was approximately 25 hours. Data checking and supervision of field staff is necessary for any observational data collection and is not necessarily any more costly for the collection of data at high spatial resolution. Working in pairs of 2, data collection staff spent an average of 10.5 minutes making observations on each face block in the neighborhood. Data input time was minimized since the data collection staff entered all observations directly into laptop and tablet computers. Four teams of data collection staff were sent out each day, and approximately 3 hours was required each day to extract and integrate the data from each laptop or tablet computer back into ArcGIS geodatabase when the staff returned from the field. This task was completed by the project supervisor and coincided with the staff supervision and data checking tasks.

## Results and Discussion

In analyzing the observational data, we were interested first in the inter-rater reliability of the data collection effort. Second, we analyze the relationships within the data via factor analysis and a comparison of the differences between face blocks that were rated as being of overall high and low quality. Last, we explored multiple geographic aggregation schemes and compare the information available from the data depending on the aggregation scheme used.

### Reliability

Inter-rater reliability was calculated using percent agreement based on the 10% sample of parcels for which 2 independent observations were recorded. For parcel-level items, agreement ranged from .66 to .998, with an average level of agreement of .91. For face block-level items, agreement ranged from .65 to .99, with an average level of agreement of .84. The results compare well with community audit measures as reported by Brownson et al. [29]. The higher repeatability for parcel level observations is likely due to the smaller observational unit and suggests that aggregates built up from parcel observations may produce more reliable results.

### Relationships within the data

Factor analysis using a principal factor method with a promax rotation was implemented to distill the parcel-level data into summary variables. Two factors with eigenvalues greater than 1 were identified, and factor loadings for the parcel items are displayed in Table 2. Items that failed to load on either factor with a loading of at least .30 were dropped. The first factor, *Aesthetics* was comprised of housing condition, peeling paint, boarded windows, unkempt lawn, fence in poor shape, and trash in yard. The second factor, *Security* was comprised of presence of barred windows and doors. The internal reliability of the factors was .61 and .83, respectively; correlation between the factors was .14, *p*<.001. Summary scores for each factor were calculated by coding each parcel-level item as 1 to represent undesirable attributes and summing the component items. This method was chosen rather than using factor loadings to preserve the intuition behind the Aesthetic and Security variables—i.e. Aesthetic is the number of undesirable attributes of a parcel. Higher scores on the *Aesthetic* factor, the primary parcel-level indicator of neighborhood condition, indicated more “eye-sore” conditions on a parcel.

**Table 2:** Factor loadings for parcel-level items.

	Factor	
Item	Aesthetics	Security
Housing condition	.39	−.05
Peeling paint	.43	.02
Broken windows	.21	−.01
Boarded windows	.30	−.08
Barred windows	.01	.75
Barred doors	−.01	.75
Uncovered crawlspace	.29	.01
Unkempt lawn	.45	.00
Fence in poor shape	.40	.03
Trash on curb	.13	.01
Trash in yard	.41	.02
Cars in yard/drive needing repair	.22	.05

The items recorded at the face-block level did not lend themselves to a data reduction method such as factor analysis. To examine the interrelations of these items, therefore, we compared the characteristics of face blocks receiving an overall rating of “desirable” with those rated “undesirable”. The results and chi square statistics (Fisher exact) are displayed in Tables 3 and 4. Desirable blocks were wider, less congested, had better maintained streets and sidewalks, more street lamps, and fewer unrestrained dogs. Additionally, desirable face blocks are more likely to contain parcels with fewer *Aesthetic* concerns (the Spearman rank correlation coefficient is −.20, *p*<.01).

**Table 3:** Differences in face block conditions for desirable vs. undesirable face blocks—Sidewalk and Street Conditions.

	Undesirable		Desirable		
	N	%	N	%	Chi-Squared
**Street width**					
Two lane (no parking)	171	60.6	401	29.6	134.55***
Two lane (parking)	83	29.4	641	47.3	
Three lanes	5	1.8	169	12.5	
Four lanes	13	4.6	135	10.0	
Other width	10	3.6	7	.5	
Not a street	0	.0	1	.1	
**Divided street**					
No	276	97.5	1139	84.0	36.32***
Yes	7	2.5	217	16.0	
**Street condition**					
Rough	93	33.1	68	5.0	207.47***
Average	184	65.5	1226	90.8	
Excellent	4	1.4	56	4.2	
**Sidewalks**					
None	52	21.3	105	8.3	37.02***
One side	27	11.1	151	12.0	
Both sides	165	67.6	1005	79.7	
**Sidewalk conditions**					
Rough	76	37.1	93	7.9	142.64***
Average	129	62.9	1044	88.6	
Excellent	0	.0	42	3.6	
**Curbs**					
None	39	17.5	47	3.7	68.20***
One side	8	3.6	29	2.3	
Both sides	176	78.9	1189	94.0	
**Alley**					
None	77	92.8	442	95.1	1.31
One side	5	6.0	16	3.4	

**p<.01 ***p<.001, Fisher exact.

**Table 4:** Differences in face block conditions for desirable vs undesirable face blocks—Aesthetics and Social Factors.

	Undesirable		Desirable		
	N	%	N	%	Chi-Squared
**Street lamps**					
None	43	16.2	85	6.4	30.00***
One	51	19.2	253	19.0	
Two or more	171	64.5	997	74.7	
**Tree coverage**					
No	207	73.4	964	71.2	.56
Yes	75	26.6	390	28.8	
**Congested**					
No	232	81.9	1268	93.7	41.88***
Yes	51	18.1	86	6.4	
**Children present**					
No	279	98.9	1339	98.9	.004
Yes	3	1.1	15	1.1	
**Adults present**					
No	251	89.0	1231	90.9	1.0
Yes	31	11.0	123	9.1	
**Unrestrained dogs present**					
No	265	94.0	1316	97.2	7.46**
Yes	17	6.0	38	2.8	

**p<.01.

***p<.001.

### Geographic aggregation results

Of key interest is how the parcel data compare to more aggregated data used in other studies. More geographically aggregated data are easier and potentially less costly to obtain; however, the aggregation may wash out key variations that occur within neighborhoods. For studies of neighborhood influences, we would argue that such variations that occur (for instance) between parcels on the same face block or consecutive streets in the same census block might be important.

To examine the range and variability of observed measures at different levels of aggregation, we aggregated the parcel-level scores for aesthetics, security and the overall desirability of each face block to geographic groupings commonly used in the literature: face block, census block, census block group, and census tract. Aggregation was achieved by taking the average of each variable over the specified geography. Geographic boundaries that are meaningful in the sense that they represent true boundaries for different neighborhood types should delineate homogeneous data groupings. One would expect the *range* of the mean values for each grouping to not decrease at higher levels of aggregation and the average mean absolute deviation (MAD) within each grouping to not increase considerably. However, as we see in Table 5, as the level of aggregation increased, the range of scores for each observed measure decreased and the average MAD increased.

**Table 5:** Descriptive Statistics of neighborhood factors at different geographic aggregations.

Observed Characteristic	Aggregation level	Mean^a^	Minimum	Maximum	Range	Mean Absolute Deviation^b^
Aesthetics	Face block	1.139	.000	5.000	5.000	.395
	Census block	1.107	.000	4.167	4.167	.481
	Census block group	1.031	.000	2.000	2.000	.688
	Census tract	1.030	.280	1.581	1.301	.778
Security	Face block	.178	.000	2.000	2.000	.212
	Census block	.194	.000	1.111	1.111	.278
	Census block group	.239	.000	.621	.621	.377
	Census tract	.226	.020	.418	.398	.368
Overall desirability	Census block	.832	.000	1.000	1.000	.126
	Census block group	.865	.574	1.000	.426	.203
	Census tract	.848	.705	1.000	.295	.240

a Values reported are the mean of the value for all groups.

b Refers to the mean of the mean absolute deviation within each aggregation.

**Figure 1:**
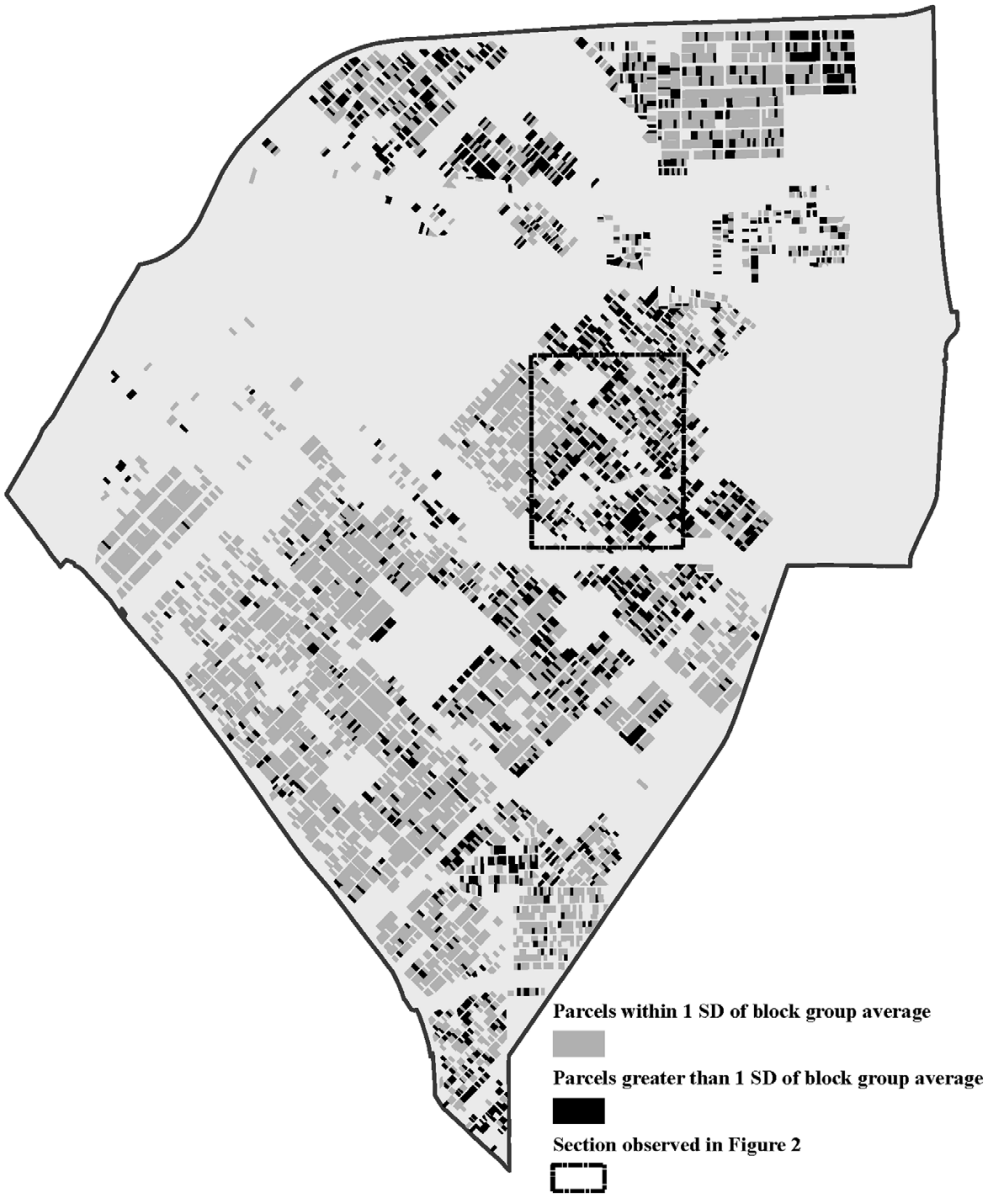
Comparison of Parcel *Aesthetic* and Block Group Average *Aesthetic*.

To further examine the impact of aggregation on the data, we compared the parcel values for *Aesthetic* to the block group average values of *Aesthetic*; results are displayed in Figure 1. Parcels shaded black have a block group average value for *Aesthetic* that differ by one or more standard deviations (one SD = 1.14) from the actual parcel value. Over 25% of the parcels are mischaracterized by the block group average value of *Aesthetic* by at least one standard deviation. This is important when considering causal relationships between individual outcomes and neighborhood. An individual may influence the level of *Aesthetic* of his/her own parcel, but may have little control over *Aesthetic* at the face block (or higher) level.

The spatial analysis may be taken a step further by computing local Moran's *I* statistics for each geographic aggregation level using ESRI ArcMap Cluster and Outlier Analysis tool in the Spatial Statistics toolbox. The local Moran's *I* is a local indicator of spatial association (LISA) and takes on statistically significant positive (negative) values when there is spatial clustering of similar (dissimilar) values [41]. The spatial clustering of data is at the heart of analyzing the influence of place on observed outcome variables such as physical activity, child development, crime or economic development. High or low degrees of spatial clustering can result in very different policy implications. For example, a block group of average neighborhood condition might in fact be composed of two distinct clusters—one with high neighborhood quality and one of low neighborhood quality. Further, spatial clustering that occurs along the boundaries between neighborhoods might result in significant edge effects that result in misleading correlative analysis.

**Figure 2:**
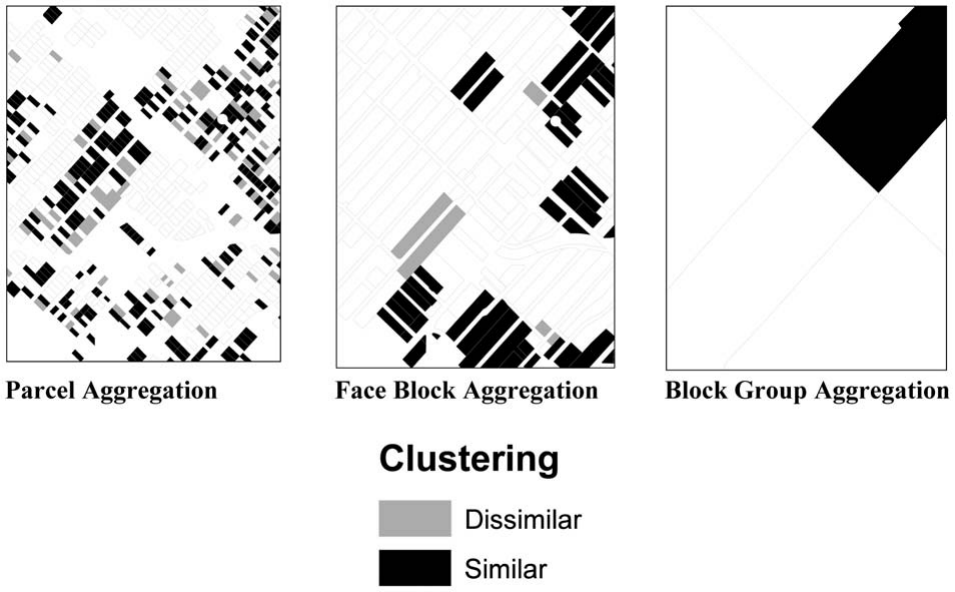
Moran's I at Parcel, Face Block and Block Group Aggregation.

Table 6 presents the percentage of geographic units with statistically significant spatial clustering at each aggregation level. The degree to which spatial clustering occurs—measured as a percentage of total geographic units–decreases considerably at higher levels of aggregation; an obvious consequence of aggregation. Figure 2 illustrates this observation by focusing on a small quadrant in a residential section of the neighborhood. This quadrant being analyzed is indicated in Figure 1. Spatial clustering of like values is indicated in black, while spatial clustering of dissimilar values is shown in grey. Un-shaded areas do not exhibit statistically significant spatial clustering. Observing only the block group level data, one would conclude that there is little spatial clustering in this section of the neighborhood, when in fact many of the parcels are spatially clustered. A similar pattern emerges throughout the neighborhood. The role of spatial clustering of observational data is very important from a policy perspective because it can provide guidance as to whether policy should tackle small concentrated areas of high concern or should be applied on a larger, less concentrated scale.

**Table 6:** Percentage of geographic units with statistically significant spatial clustering at each aggregation level.

Aggregation Level	Percent with Significant Moran's I
Census block group	22%
Face block	30%
Parcel	40%

### Predictive Utility for Health Outcomes

Table 7 presents the results of a simple demonstration of the utility of the Aesthetics score obtained from our neighborhood observational data at predicting self-reported health status. A univariate ordered logit model was applied to determine the relationship between self-reported health status (*M* = 2.78, *SD* = 1.07) and *Aesthetics* at varying levels of geographic aggregation. The odds ratios and corresponding 95% confidence intervals are reported in Table 7.

**Table 7:** Relationship between neighborhood condition and health status at varying levels of geographic aggregation.

	Parcel	Faceblock	Census Block	Census Block Group	Census Tract
**Aesthetic OR**	1.103*	1.152+	1.054	1.123	1.532***
**95% CI**	(1.004–1.212)	(0.996–1.333)	(0.920–1.207)	(0.926–1.362)	(1.194–1.967)

*** p<0.001, ** p<0.01, * p<0.05, + p<0.10.

*Aesthetics* at the census tract level has the highest odds ratio and statistical significance. However, we find that no statistically significant relationship can be found at the census block group aggregation. The relationship reappears at the faceblock and parcel level. These results illuminate the need to improve policy research by exploring different aggregation schemes and the necessity of a method for neighborhood observations that has high spatial resolution so that the next step can be taken: understanding why the relationships change with spatial aggregation. This perhaps overly simplistic demonstration does not necessarily indicate that one aggregation level is best for all cases. Instead, this simple example indicates that the estimated associations vary depending upon geographic aggregation, and the research question should dictate the aggregation level used—or put differently changing the aggregation *changes* the research question being answered. For example, if we want to know which low income census tracts to target for a public health information campaign, then tract level is appropriate. However, if we want to know how to best invest public funds to improve public health through neighborhoods, and policy makers must choose between specific small-scale projects, the aggregation level used should match the scope of the projects in question. Further, when theory does not clearly define a superior aggregation level, results should be evaluated with consideration for their robustness to alternative geographic specifications.

### Conclusions

This paper presents and applies a new methodology for obtaining community audit data of neighborhood condition at the smallest geographic unit, the parcel. Our instrument for systematic neighborhood observations presents a high degree of inter-rater reliability, factor analysis revealed logical relationships within the data, and the factors derived from the data were predictive of health status. Of key interest to our work was assessing the costs and benefits of obtaining data at such a high spatial resolution.

Obtaining observation data is not without costs. There are some significant up front costs that are required for observational data collection at any geographic aggregation level—GIS expertise, software and hardware for data entry and organization, and supervision for project staff. The primary additional cost of obtaining observational data at high spatial resolution is time. Our systematic neighborhood observations averaged 10.5 minutes per face block. This compares favorably with other instruments designed for observational data collection at lower geographic resolution. The average time for observations for these lower-resolution instruments ranged from 10.6 to 20 minutes/segment [29]. This suggests that collecting parcel level data is not overly expensive or time-consuming if face block or street segment observations are already being conducted once the requisite GIS is in place.

However, exploratory analysis of the data reveals that much detail is lost when the data are averaged to higher levels of aggregation. Therefore, parcel level data may provide many benefits. Higher levels of aggregation result in less variability among observations and lack of resolution when identifying statistically significant spatial clustering. This lack of resolution may be a key hindrance in uncovering correlative relationships between neighborhood condition and observed outcome measures when the parcel level attributes are not observed.

Another key advantage to obtaining small area geo-referenced data is the ability to explore different aggregations, both a variety of census geographies and also many other more flexible aggregation schemes such as the neighborhood condition within 250, 500 or 1000 feet of a parcel. This allows an array of research questions to be answered without the limitation of the aggregation scheme available in the data and the answers to the research question can provide more specific policy recommendations about the geographic scale of policy interventions. For example, child physical activity is likely influenced by neighborhood conditions based upon the routes a child might take through a neighborhood. The route through the neighborhood from the child's home to school dictates the relevant section of the neighborhood that matters for the decision of whether to walk or drive to school. The quality of this route may be distorted if proper geographic aggregation is not employed. Further, our results indicate variation in associative relationships between health status and *Aesthetics* due to geographic resolution. These differences allow the results to inform policy to a much greater extent because the relationship between individual parcel upkeep, near neighborhood condition and more distant neighborhood condition can be explicitly explored.

However, we should note that the utility and necessity of data at high spatial resolution will vary depending upon the research question being analyzed. Broad policy questions may effectively be answered with data at lower resolution. Further, analysis that requires observational data over large areas (e.g. counties, states, etc) are likely not practical applications for parcel-level data because of the time required for a sufficient amount of sampling for coverage in these larger areas. Nevertheless, the methodology and associated analysis presented here clearly indicate that collecting affordable high quality neighborhood data at the micro level is possible. Further, there are significant differences in how a particular location is classified in terms of neighborhood condition depending upon the geographic aggregation scheme used. In the absence of a sound, tested theoretical basis for defining a particular geographic aggregation, it is important to analyze the effect that geographic aggregation has on the correlative relationships being studied. Otherwise one cannot understand how the results are being affected by the particular geographic definitions used for the study. The systematic neighborhood observation methodology presented here demonstrates how parcel level data can be collected at minimal additional cost to answer these important questions.
